# The impact of policy incentives and value perception on rural residents’ clean heating behavior: Evidence from northern China

**DOI:** 10.1371/journal.pone.0321936

**Published:** 2025-04-17

**Authors:** Yuxuan You, Xia Xu, Guanqiu Yin

**Affiliations:** 1 College of Economics and Management, Shenyang Agricultural University, Shenyang, Liaoning, China; 2 College of Economics and Management, Nanjing Agricultural University, Nanjing, Jiangsu, China; Wuhan Institute of Technology, CHINA

## Abstract

Implementing energy transition in rural areas is crucial for China to achieve its low-carbon transition in energy consumption and dual-carbon goals. This study aimed to elucidate policy effects and further analyze the mediating effect of value perception to provide a reference for building a long-term rural energy transition mechanism. We constructed a “policy incentives–value perception–behavior” theoretical analysis framework and used survey data collected from residents of northern China. A logit model was employed to empirically test the effects of advocacy, demonstration, and subsidy policies on residents’ clean heating behavior. We used a mediation effect model to examine the mediating effects of economic, functional, social, and emotional value perceptions. The results showed that all three policies significantly positively impacted residents’ clean heating choices, with subsidy policies exerting the best effect. These findings suggest that implementing policy incentives can influence residents’ behavior by enhancing their value perceptions. However, different types of policies may act through distinct pathways. Compared with previous studies that focused solely on the impact of policy or value perception on clean heating behavior, this study explored their interactive relationship and found that external policy incentives can be transformed into internal driving forces. Therefore, value perception should be considered during policy formulation to build a long-term mechanism for promoting energy transition in rural areas.

## Introduction

Rural energy transition is related to sustainable environmental, economic, and social development. As early as the 1960s–70s, developed countries had completed the transition from bulk coal to natural gas heating in winter. However, in the rural areas of northern China, scattered coal combustion has remained the mainstay owing to dispersed settlements and a lack of infrastructure [1]. This heating method causes severe winter pollution [2,3]. Clean energy (cleaner coal, gas, electricity, geothermal, solar, etc.) is generally more efficient and causes less pollution [4–6]; however, owing to the relatively high cost of clean energy and the lack of policy interventions, rural areas using clean heating accounted for less than 20% at the end of 2016 [7]. To promote the energy transition, the Chinese government issued “Planning of Winter Clean Heating in North China.” By 2022, China had invested 107.1 billion yuan in five clean heating batches comprising 88 pilot cities. However, because of the excessive economic burden, inexperience with electric heating equipment, and other problems, residents showed low willingness to adopt clean heating. Consequently, the clean heating penetration rate is extremely low, and loose coal is reburned in many rural areas [8,9]. An understanding of the factors that influence residents’ energy demand is necessary to achieve a rural energy consumption transition.

Given the intense concerns regarding air pollution and carbon emissions, clean heating in the residential sector has become a trending topic. Many studies have established the importance of residents’ family endowments, individual characteristics, and attitudes as internal influencing factors on clean heating behavior (CHB). Early studies analyzed the impact of household income on the structure of energy consumption, as represented by the energy ladder theory, which suggests that household energy choices move from primitive to transitional as income increases. Based on this theory, studies have compared consumption structure characteristics and energy transition speed in rural and urban areas [10,11]. Rural residents’ willingness to pay for clean energy increases as their income rises [12], and higher-income households prefer cleaner heating choices [13,14]. Off-farm work can significantly increase rural household income and, ultimately, energy use [15,16]. Choosing clean heating is also related to factors such as age, gender, and education [17–20]. In addition to these socioeconomic factors, rural residents’ decisions depend on their attitudes, and value perception is the most fundamental reason for the formation of farmers’ attitudes. A household survey conducted in Greece showed a significant relationship between household willingness to adopt new heating technologies and people’s environmental awareness and perceptions [20]. Fan and Huo. (2021) [21] assessed the likelihood of rural residents’ participation in CHB in terms of their perceptions of ease of use, convenience, and other characteristics of clean heating technologies. Additionally, Xiong et al. (2021) [22] evaluated rural residents’ environmental perceptions and explored the role of their perception intensity in heating behavior.

The use of clean energy is a common externality issue. Governments in Europe and Japan have examined residents’ use of clean energy and its economic effects to create reasonable policy incentives [23,24]. For Chinese cities in the Beijing–Tianjin–Hebei area, the higher the financial support for clean heating, the higher the clean heating rate [25]. Some studies have analyzed how to set reasonable subsidy standards. Li et al. (2021) [26] constructed a model containing clean heating options, rekindling rate, and clean heating costs, and measured the optimal subsidy standard and amount. Yan et al. (2020) [27] used the minimum data method to incorporate the pollution emissions reduction target and heating subsidy standard into the same model and found that a monthly compensation of 9 yuan/m^2^ could reduce pollutant emissions by 96.7%. Other scholars have argued that fuel subsidization is unsustainable [28,29]. Some studies have actively explored the effects of policies such as demonstrations and technical support [26,30,31]. However, specific problems were found in policy implementation, such as problems with economic affordability among poor farmers, high clean energy costs, low sustainability of subsidies, and incompleteness of subsidy policies [21]. The gap between subsidies in China and the population’s actual needs is approximately 50% [32]. Households are also likely to return to coal heating within 3 years following the cessation of the local financial support policy [33].

In summary, existing studies have analyzed the factors influencing residents’ CHB based on both intrahousehold and policy factors. However, the literature has several shortcomings. First, it focuses mainly on subsidy policies. As shown above, their effect is limited. When many subsidies are available, the local financial burden becomes heavy. Thus, a multidimensional policy mix is required. Second, most previous studies separately explored the impact of value perception and policy incentives on CHB, while neglecting their interactive relationship. Policies have alternative and complementary functions to marketing, and if relevant policies can be assimilated into residents’ value perceptions, they will contribute to building a long-term promotion mechanism for rural energy transition. Third, these studies’ analyses used relatively little household-level research data. Most relied upon macrodata or case studies to evaluate policy effects. Residents are both implementers and beneficiaries of clean heating, and a better understanding of heating energy use at the household level is needed.

In view of this, the present study obtained the survey data of rural residents in northern China, constructed a theoretical analysis framework based on the perspective of external incentives and endogenous driving forces, employed a logit and mediation effect model to test the impact of policy incentives on residents’ CHB, and further explored the mediation effect of value perception to provide a theoretical basis for improving clean heating policy and promoting rural energy transition. This study contributes to the existing literature in three ways. First, in addition to examining subsidy policies, we analyzed the influence of advocacy and demonstration policies on CHB. Second, we constructed a theoretical analysis framework comprising “policy incentives–value perception–behavior” to answer the questions of whether and how external policy incentives can become internal drivers. Finally, combining both policy and value perception in the model helped to avoid the endogeneity problem caused by the omission of variables, and replacing the policy variable with whether a city was a pilot city improved the reliability of the results.

## Theoretical analysis

### Impact of policy incentives on CHB

CHB has external effects. If the economic cost greatly exceeds the direct benefits generated during implementation, rural residents will be insufficiently motivated to adopt clean heating [34]. Therefore, when promoting a heating strategy, the government should maximize the internalization of the externality of CHB through a series of policy tools to promote the adoption of clean heating [35]. Currently, the main types of clean heating policies in northern China are advocacy, demonstrations, and subsidization. Advocacy policies refer to the government’s promotion and popularization of clean heating, which can increase residents’ awareness of the benefits of clean heating and clean heating products [36]. Demonstration policies refer to the government’s adoption of relevant agricultural demonstration projects to dispel rural residents’ concerns about using clean heating products and transform their wait-and-see attitude into heating behavior [37]. Finally, residents reap direct benefits through government-implemented economic compensation and reward measures, which enhance their enthusiasm for adopting clean heating [38]. Therefore, we proposed the following hypothesis:

**Hypothesis 1 (H1)**: Policy incentives significantly positively impact the CHB of rural residents in northern China.

### Mediating effect of value perception

According to the theory of behavioral economics, a person’s rational or irrational behavior is affected by their cognitive level and perception [39,40]. Value perception is a subjective balance between effort and gain based on existing cognition, and is thus a key variable in exploring individual willingness and behavior. When rural residents pay to adopt clean heating, they gain benefits such as convenience of life, environmental improvement, and maintenance of physical health. However, they also face risks and costs including increased heating costs, the maintenance costs associated with heating equipment, and an unstable energy supply [41]. Hence, when rural residents are deciding whether to adopt clean heating, they evaluate its benefits and risks, thus forming a subjective perception of value.

Value perceptions can be divided into economic, social, functional, and emotional. Additionally, the establishment of rural residents’ value perception is influenced not only by personal characteristics but also by external factors [ 42,43] including government incentives and neighborhood behaviors. The government employs affirmative incentives such as subsidies, demonstrations, and advocacy to enhance rural residents’ perceptions of the value of clean heating, helping them recognize its long-term advantages. As policy incentives increase, residents’ appreciation of the value of clean heating strengthens steadily, thereby fostering a greater inclination toward adopting a clean heating method. Therefore, the policy not only directly impacts rural residents’ CHB but also indirectly affects their behavior by changing their value perception. Therefore, we proposed a second hypothesis:

**Hypothesis 2 (H2):** Policy incentives indirectly affect CHB by influencing residents’ value perception.

**Fig 1:**
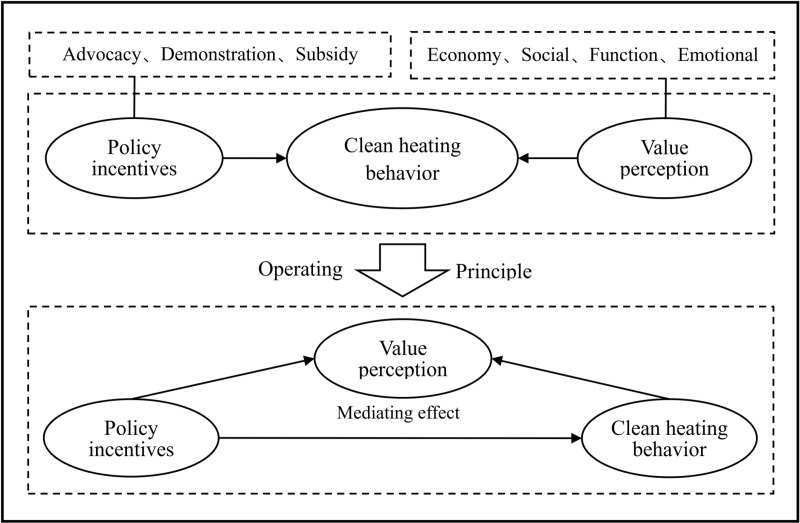
Theoretical framework.

Based on the above theoretical analysis, we constructed a framework for policy incentives, value perception, and clean heating decision-making among rural residents in northern China (Fig 1).

## Materials and methods

### Data source

We obtained the data used in this study from an online survey conducted in January–March 2023 among rural residents of northern China. We obtained ethical clearance from the Shenyang Agricultural University (reference number: SYAU2023012309). The survey was conducted in the northern regions designated in the “Clean Winter Heating Plan in Northern China” (referred to as the “Plan” hereinafter); these include 15 provinces and cities: Heilongjiang, Jilin, Liaoning, Inner Mongolia, Beijing, Tianjin, Henan, Hebei, Shanxi, Shandong, Shaanxi, Ningxia, Gansu, Qinghai, and Xinjiang. To collect larger samples from different areas, we distributed an online questionnaire to rural residents for voluntary and anonymous completion. The questionnaire included a specific item inquiring about the heating methods respondents used throughout winter. To ensure the inclusion of suitable participants, sample selection involved a blend of stratified and snowball sampling. Initially, we categorized the northern region into pilot and non-pilot areas in accordance with the guidelines outlined in the “Plan.” Subsequently, 50 cities were randomly selected from both areas. Approximately 10 rural residents who practiced independent winter heating were selected from each city to complete the questionnaire. Following a snowball sampling approach, participants were asked to provide information about other potential survey candidates. To improve the sample’s representativeness, we administered supplementary surveys based on an analysis of the main survey respondents’ distribution across different areas. The supplementary surveys were conducted to adjust the sample sizes in the pilot and non-pilot areas to achieve a balanced distribution and ensure an adequate number of samples from representative regions. During the supplementary survey phase, the northeastern region sample size increased owing to the region’s low winter temperatures and long periods requiring heating. To reduce the potential sample selection bias inherent in snowball sampling, we selected the first batch of survey participants from among rural residents with diverse occupations. To improve the quality of the questionnaire responses, we included logical analysis questions to determine whether the respondents had answered seriously and whether the data provided were logically consistent. Moreover, geographical restrictions were imposed based on respondents’ IP addresses to prevent multiple responses from the same individual. Questionnaires with excessively short completion times, responses that were inconsistent with the research area, and errors and omissions were filtered to ensure the accuracy, validity, and representativeness of the research data. We collected 546 questionnaires from respondents in 104 cities, of which 57 were pilot cities (54.81%), and 47 were non-pilot cities (45.19%). The questionnaire covered rural residents’ personal and household characteristics, clean heating features, and value perceptions. Samples with missing data, abnormal values were excluded. Finally, we obtained 477 valid questionnaires, resulting in an effective response rate of 87.36%.

### Model setting

#### Logit model.

Given that the adoption of clean heating among rural residents in the northern regions represents a typical binary decision-making scenario, we employed a binary logit model to analyze various influential factors. We established the regression model as follows:


$$Y_{i}=\alpha_{0}+\alpha_{1}X_{i}+\alpha_{2}C_{i}+\varepsilon$$ (1)

$$P=F\left(\text{Y}\right)=\frac{1}{1+e^{-Y_{i}}}$$ (2)

where $X_{i}$ represents policy incentives divided into advocacy, demonstration, and subsidy policies; $C_{i}$ is the control variable; $Y_{i}$ is CHB; and *P* is the probability of CHB. The binary logit expression is:


$$ln\left(\frac{P}{1-P}\right)=\alpha_{0}+\alpha_{1}X_{i}+\alpha_{2}C_{i}+\varepsilon$$ (3)

#### Mediating effect model.

We constructed a mediator model to test the mechanism of policy incentives’ effect on CHB to determine whether they promote CHB through value perception. Based on a previous study [44], the mediator model was constructed as follows:


$$Y_{i}=a_{1}+aX_{i}+\delta C_{i}+\varepsilon_{1}$$ (4)

$$M_{i}=a_{2}+bX_{i}+\delta_{2}C_{i}+\varepsilon_{2}$$ (5)

$$Y_{i}=a_{3}+a^{\prime }X_{i}+cM_{i}+\delta_{3}C_{i}+\varepsilon_{3}$$ (6)

where $M_{i}$ is the mediator variable, and *ε* is the perturbations term. The mediating effect was tested in three steps. Step 1 entailed regressing Model (4). A significant coefficient *a* indicated that policy incentives affected residents’ CHB, and we proceeded to the next step. Step 2 entailed regressing Model (5). A significant coefficient *b* indicated that policy incentives affected value perception, and we proceeded to the next step. Step 3 entailed regressing Model (6). Significant coefficients *a*^′^ and *c* indicated that the impact of policy incentives on rural residents’ CHB was at least partially realized through their value perceptions. If *a*^′^ was not significant in Model (6), value perception showed a full mediating role. Furthermore, *a* in Model (4) represents the total effect, b×c refers to the mediating effect, and *a*^′^ is the direct effect. Calculating the proportion of the direct and mediating effects in the total effect facilitated a comparison of the degree of the direct and mediating effects.

### Variable selection

#### Dependent variable.

We investigated rural residents’ CHB, that is, whether residents adopted clean energy sources with ultra-low emissions for heating purposes, such as natural gas, electricity, geothermal energy, biomass, solar energy, or clean coal. If a resident adopted clean heating, the assigned value of the dependent variable was 1; if a resident did not adopt clean heating, the value of the dependent variable was 0.

#### Core independent variable.

The core independent variable was policy incentives in the context of clean heating policies in rural northern China. We categorized policy incentives into three types: advocacy, demonstration, and subsidy. We measured advocacy policy implementation by assessing whether the government advocated clean heating through the provision of related technical information. We measured demonstration policy implementation by determining whether a clean heating demonstration project existed near a given village. We measured subsidy policy implementation by asking whether residents received clean heating subsidies. A relevant policy was assigned a value of 1, and 0 otherwise.

#### Mediator variable.

Based on Sweeney’s (2001) [45] value perception measurement scale and considering the distinctive attributes of clean heating products, we selected four value perception dimensions. We measured economic value perception using the following items: “The price of clean heating is reasonable,” “I have been treated well in terms of clean heating subsidies,” and “Clean heating is worthwhile.” We measured social value perception using the items “Clean heating gives me more topics to discuss when interacting with others,” “Clean heating helps me create a positive impression on others,” and “Clean heating enhances my confidence in social situations.” We measured functional value perception using the following items: “Clean heating improves the speed and convenience of heating,” “Clean heating reduces the occurrence of respiratory illnesses,” and “Clean heating provides me with diverse heating options.” We measured emotional value perception using the items “I feel an urge to use clean heating products,” “I enjoy using clean heating products,” and “Clean heating products contribute to a positive perception of my overall life satisfaction.” All value perceptions factors were assessed on a 5-point Likert scale.

#### Control variables.

We used three categories of control variables: individual characteristics of the household head including variables such as gender, age, education, and social connections; family characteristics such as the number of family members, heating area (in m^2^), presence of village cadres within the household, engagement in non-agricultural employment, and total household income; and external factors such as the demonstration effect and herd mentality, which influence residents’ heating behavior during policy implementation. Consequently, we incorporated the neighborhood effect factor. Table 1 lists the variables’ meanings and descriptive statistics.

**Table 1:** Definitions of variables and descriptive statistics.

Variables	Definition	Mean	SD
Clean heating behavior	Whether rural residents adopted clean heating behavior (yes = 1, no = 0)	0.583	0.494
Advocacy policy	Whether the government promotes clean heating behavior (yes = 1, no = 0)	0.568	0.496
Demonstration policy	Whether the village has a clean heating demonstration project (yes = 1, no = 0)	0.509	0.500
Subsidy policy	Whether residents receive clean heating subsidies (yes = 1, no = 0)	0.509	0.500
Value perception	Whether residents perceive economic, social, functional, and emotional value (1= Strongly disagree, 2 = Disagree, 3 = Not sure, 4 = Agree, 5 = Strongly agree)	0.718	0.171
Gender	Resident’s gender (male = 1, female = 0)	0.465	0.499
Age	Resident’s age (1 = 18–25 years, 2 = 26–45 years, 3 = 46–65 years; 4 = 65 years and above)	2.122	0.851
Education level	Resident’s highest education level (1 = elementary school and below; 2 = junior high school; 3 = high school; 4 = college and above)	3.266	0.929
Social network	Number of cellphone contacts (1 = 0–29, 2 = 30–59, 3 = 60–99, 4 = 100–149, 5 =150–199, 6 = 200 and above)	3.417	1.613
Family size	Number of family members in household (1 = 1–2, 2 = 3–4, 3 = 5–6, 4 = 7 and above)	2.082	0.799
Heating area	Household winter heating area (1 = less than 30 $m^{2}$; 2 = 30–60 $m^{2}$, 3 = 60–90 $m^{2}$, 4 = 90–110 $m^{2}$, 5 = 110 $m^{2}$ and above)	2.987	1.128
Cadre	Whether someone in the family is a village cadre (yes = 1, no = 0)	0.237	0.426
Non-farm employment	Whether the family includes laborers working outside the home (yes = 1, no = 0)	0.553	0.498
Household income	Annual household income (1 = less than 10,000; 2 = 10,000–30,999; 3 = 40,000–79,999; 4 = 80,000–119,999; 5 = 120,000–159,999; 6 = 160,000 and above)	3.201	1.491
Neighborhood effect	Whether neighbors implement clean heating behavior (yes = 1, no = 0)	0.501	0.501

## Results

### Impact of policy incentives on CHB

The efficacy of policy implementation depends on its target group’s responsive actions. Positive audience responses are often received when good policies are implemented effectively, whereas negative responses may arise otherwise. Table 2 presents the estimated results of the logit model used to examined the impact of policy incentives on CHB. Models (1)–(3) were employed to assess the impacts of advocacy, demonstration, and subsidy policies on CHB. These models’ estimated coefficients were 1.525, 1.348, and 2.363, respectively, and all were statistically significant at the 1% level. Advocacy policies significantly impacted residents’ CHB as evidenced by the Model (1) results. This notable effect can be attributed to the proactive influence of clean heating promotion policies, which enhance public awareness by providing information and guidance, thereby facilitating the adoption and widespread implementation of clean heating. The greater the number and intensity of government policy promotion methods, the higher the likelihood of rural residents continuing to adopt clean heating under the influence of policy promotion [46]. Model (2) revealed that demonstration policies also significantly influenced CHB. This effect could be credited to demonstrations’ efficacy at showcasing successful instances of clean heating, offering consultation, training, and guidance to the public, and demonstrating the feasibility and tangible benefits of clean heating. These initiatives enable rural residents to comprehend and select appropriate clean heating technologies and equipment, thereby fostering the adoption of clean heating methods. The results of Model (3) indicated a significant impact of subsidy policy on rural residents’ CHB, underscoring that subsidy policies effectively stimulate CHB by directly reducing the costs associated with its usage and alleviating the economic burden of adopting clean heating technology. In summary, policy incentives directly shape residents’ CHB, validating H1.

**Table 2:** Impacts of policy incentives on rural residents’ CHB.

Variables	Model (1)		Model (2)		Model (3)	
Coef.	S.E.	Coef.	S.E.	Coef.	S.E.
Advocacy policy	1.525***	0.254				
Demonstration policy			1.348***	0.251		
Subsidy policy					2.363***	0.284
Gender	-0.084	0.255	-0.088	0.254	-0.139	0.273
Age	0.360**	0.168	0.338**	0.166	0.251	0.177
Years of education	0.155	0.156	0.113	0.156	0.096	0.169
Social network	-0.083	0.079	-0.106	0.078	-0.165*	0.086
Family size	0.115	0.157	0.066	0.159	-0.076	0.169
Non-farm employment	0.646**	0.253	0.486*	0.249	0.521*	0.267
Household income	0.051	0.091	0.071	0.090	0.089	0.097
Cadre	0.819**	0.309	0.732**	0.305	0.168	0.331
Heating area	-0.044	0.116	-0.041	0.116	0.045	0.124
Neighborhood effect	2.353***	0.256	2.317***	0.255	2.033***	0.272
Constant term	-3.266***	0.906	-2.654***	0.882	-2.380***	0.930
Sample size	477		477		477	

*, * *, and * * * indicate significance at the 10%, 5%, and 1% levels, respectively.

### Mediating effect of value perception

The analytical results confirmed the significant positive impact of policy incentives on the CHB of rural residents in northern China. Subsequently, we employed Sobel mediation analysis, referring to the mediating effect model, to explore the mechanism through which policy incentives influence CHB and whether policy incentives further impact it through value perception. Table 3 presents the results.

**Table 3:** Estimation results for the mediating effect.

Variables	Model (1)	Model (2)	Model (3)	Model (4)	Model (5)	Model (6)	Model (7)	Model (8)
Advocacy policy	0.237*** (0.038)	0.243*** (0.038)						
Demonstration policy			0.197*** (0.040)	0.205*** (0.039)	0.213*** (0.039)	0.208*** (0.040)		
Subsidy policy							0.387*** (0.041)	0.397*** (0.040)
Economic value	0.363*** (0.093)		0.340*** (0.096)				0.187** (0.092)	
Social value		0.295*** (0.083)		0.275*** (0.085)				
Functional value					0.245** (0.097)			
Emotional value						0.269*** (0.096)		0.218** (0.090)
Control variables	Controlled	Controlled	Controlled	Controlled	Controlled	Controlled	Controlled	Controlled
R-squared	0.432	0.428	0.415	0.412	0.407	0.409	0.482	0.483
Sample size	477	477	477	477	477	477	477	477
Goodman- (Aroian)	0.020**	0.014*	0.030***	0.022**	0.014*	0.018**	0.025*	0.015*
Proportion of total effect that is mediated	0.079	0.056	0.133	0.098	0.063	0.081	0.061	0.036

*, * *, and * * * indicate significance at the 10%, 5%, and 1% levels, respectively. Due to space constraints, the estimated results of formulas (4) and (5) and the results of the insignificant mediating effect are omitted. Descriptive statistics for the interquartile range are included in the supporting information section.

Estimation models (1 and 3), pertaining to the mediating effect of advocacy policies, produced coefficients of 0.363 and 0.295, respectively, that were significant at the 1% level. These results indicated that economic and social value perceptions mediated advocacy policies’ impact on rural residents’ CHB, and the proportion of the total effect mediated was 0.079 and 0.056, respectively. This finding implies that advocacy policies facilitate the adoption of clean heating by enhancing residents’ economic and social value perceptions. Such policies effectively communicate the importance and benefits of clean heating to the public through advertising, promotional activities, education, and training. Consequently, rural residents have become increasingly aware of the social issues associated with traditional heating methods and have developed a clearer understanding of alternative heating options, thereby encouraging their adoption of clean heating. Moreover, the implementation of policies such as clean heating subsidies, tax reductions, loans, and rewards has elevated rural residents’ perceptions of the economic value of clean heating. They have recognized that the benefits derived from clean heating outweigh the costs incurred, and this realization has intensified their motivation to embrace clean heating technology.

Estimation models (3–6), used to examine the mediating effects of demonstration policies, produced coefficients of 0.340, 0.275, 0.245, and 0.269 for economic, social, functional, and emotional value perception, respectively, all of which were significant at the 1% and 5% levels. These results indicated that economic, social, functional, and emotional value perceptions partially mediated demonstration policies’ impact on CHB. The corresponding mediating effects were 0.133, 0.098, 0.063, and 0.081. Demonstrations inspire and direct the public by presenting successful instances of clean heating. This approach enables rural residents to gain a more intuitive understanding of the convenient, rapid, efficient, and health-promoting energy clean heating can deliver, thus fostering their appreciation of its functional value. Furthermore, as rural residents witness their neighbors, friends, communities, or others effectively embracing clean heating technology and experiencing tangible benefits, their emotional connection to clean heating is reinforced, thereby stimulating its adoption. Additionally, the promotion effect demonstration policies achieve contributes to the formation of social recognition and consensus regarding clean heating, and, as a growing number of individuals adopt clean heating technology, CHB becomes increasingly synonymous with social responsibility and environmental awareness [47]. This emerging social identity and consensus create positive social pressure among residents, amplifying their perception of the social value of clean heating from an external perspective. Consequently, residents are incentivized to embrace CHB to attain social recognition and align themselves with social expectations.

Lastly, we used Models (7) and (8) to examine the mediating effects of subsidy policies. The impact coefficients for economic and emotional value perception were 0.187 and 0.218, respectively, and both were significant at the 5% level. These results highlighted the partial roles of economic and emotional value perceptions in mediating subsidy policies’ impact on residents’ CHB, thus contributing to the overall mediating effect. The mediating effect values were 0.061 and 0.036, respectively. This finding suggests that rural residents demonstrate a rational preference for seeking benefits and avoiding risks when making decisions regarding the adoption of clean heating. Subsidy policies commonly manifest as direct financial subsidies, tax exemptions or deductions, and incentivized energy pricing. As rural residents perceive greater benefits and lower risks, their perception of the value associated with clean heating increases. Consequently, residents are more inclined to adopt clean heating methods.

### Robustness test

The benchmark regression model measured the core independent variable based on residents’ subjective evaluations. Those who adopted clean heating were more concerned about relevant policies, and their evaluations reflected actual policy levels, whereas residents who had not adopted clean heating were likely to underestimate the policy level. An endogeneity issue may have arisen because of this causal relationship. Therefore, we conducted a robustness test wherein we replaced the policy incentive variables with whether the respondent household was in a pilot city. The policy implementation level is higher in pilot versus non-pilot cities. We referenced the “Plan” to determine the classification of pilot and non-pilot cities.

The control variables remained consistent, and the estimated results after replacing the explanatory variables are shown in Table 4. Clean heating pilot city residents showed a higher probability of adopting clean heating, with significance at the 1% level. This finding is consistent with the previously reported analysis results, thereby demonstrating the robustness of the empirical results.

**Table 4:** Robustness test results.

Variables	Coeff.	S.E.	T	P	[95% Confidence	Interval]
Whether located in a pilot city	1.139***	0.245	4.64	0.000	0.658	1.620
Gender	0.030	0.250	0.12	0.906	-0.461	0.520
Age	0.345**	0.168	2.05	0.040	0.016	0.673
Education level	0.172	0.160	1.08	0.280	-0.140	0.485
Social network	-0.100	0.077	-1.31	0.191	-0.251	0.050
Family size	0.159	0.159	1.00	0.317	-0.153	0.471
Non-farm employment	0.487**	0.247	1.97	0.048	0.003	0.970
Household income	0.045	0.089	0.50	0.615	-0.130	0.220
Cadre	0.614**	0.303	2.03	0.043	0.021	1.208
Heating area	-0.041	0.114	-0.36	0.717	-0.265	0.183
Neighborhood effect	2.551***	0.252	10.11	0.000	2.056	3.045
Constant term	-2.991***	0.903	-3.31	0.001	-4.762	-1.221
Mean dependent variable		0.583		SD dependent variables		0.494
Pseudo R-squared		0.324		Number of observations		477
Chi-square		210.255		Prob> chi^2^		0.000
Akaike crit. (AIC)		461.863		Bayesian crit. (BIC)		511.873

*, * *, and * * * indicate significance at the 10%, 5%, and 1% levels, respectively

## Discussion

The results showed that the impacts of advocacy, demonstration, and subsidy policies on CHB were positive and significant at the 1% level, with coefficients of 1.525, 1.348, and 2.363, respectively. These findings suggest that advocacy, demonstration, and subsidy policies effectively promote clean heating, with subsidy policies being the most effective. The findings are consistent with those of previous studies. Jin et al. (2022) [2] showed that policy incentives can reduce relative costs and increase people’s ability and willingness to pay to adopt clean heating. However, clean heating technology has not yet matured, and equipment investment and maintenance costs are high [38]. The unit heating costs of electricity and natural gas are higher than that of scattered coal, and the economic cost is a constraint for residents considering clean heating [1,14]. Chen and Mu. (2022) [48] reported similar results indicating that, compared with other types, subsidy policies had the best effect on farmers’ adoption of water-saving technologies. These consistent findings conﬁrm that policy incentives are not monolithic; rather, they must be implemented in combination with various measures. Therefore, policymakers should focus on subsidy policies and strengthen advocacy and demonstration policy implementation to reduce the financial burden and improve effectiveness.

Policy incentives can increase value perception and ultimately influence residents’ CHB. Regardless of the choice of advocacy, demonstration, or subsidy policies, the main purpose is to use certain instruments to directly or indirectly transmit information and attributes to guide residents’ behavior. Most studies have analyzed the impact of policy incentives or value perceptions on CHB [20–22,25,31]. Some have explored the moderating effect of their interaction on behavior [48,49]. This study incorporated both in the same analytical framework and revealed value perceptions’ mediating effect. Policy incentives can affect residents’ CHB by influencing their value perception. This finding suggests that external policy incentives can be transformed into internal driving forces. Therefore, to build a long-term mechanism, value perceptions should be considered when formulating policies. Doing so would improve the low clean heating adoption rate in rural areas and help to alleviate the problem of reburning loose coal.

The action paths for different policy types differ. Our results showed that advocacy policies influenced CHB through perceptions of economic and social value. Demonstration policies promoted CHB by increasing residents’ perceptions of economic, social, functional, and emotional value. The paths of economic and emotional value perceptions in relation to subsidy policies were significant. The results also showed that increased economic value perception was a common path for all three policies. However, demonstration policy had the most comprehensive path. The long-term, stable economic and environmental value of clean heating is usually difficult for residents to perceive, thus weakening the positive effects of policy incentives. Through demonstrations, residents can feel the effects of applying clean heating technology, and these feelings comprehensively improve their value perceptions. A study that analyzed the impact of policy tools on farmers’ behavior in conservation farming on black soil reached a similar conclusion [50].

This study makes an important theoretical contribution. Planned behavior theory suggests that an individual’s behavior depends mainly on attitudes and that value perception is the basis of one’s attitudes. According to perceived value theory, an individual’s behavior is the result of their comparative weighing of benefits and risks. Motivation theory postulates that behavior is influenced not only by internal factors but also by external ones such as government policies. We combined these theories and constructed a theoretical analysis framework of “policy incentive–value perception–behavior,” which we paired with empirical analyses. Doing so broadened the perspective of the analysis of related issues and yielded an important contribution to the existing research. This study also makes a practical contribution. Government policies not only directly influence the clean heating choice but also assimilate into value perception. This finding is a useful reference for improving rural energy transition policies and building long-term promotion mechanisms.

However, this study has several limitations. First, we conducted a random sample survey in the form of questionnaire that was distributed online. Although we covered many districts, including pilot and non-pilot districts, the sample size was not balanced across districts, and detailed information such as specific policy content was not considered. Given the lack of continuous observations and survey data in existing studies, future studies should conduct analyses based on panel data obtained from indoor surveys. Second, this study did not consider other external factors such as regional resource endowments, energy prices, and equipment installation prices. To avoid advocating “one policy for all,” differentiating the types of clean heating to empirically test for differing policy effects is necessary in further research to provide a basis for differentiated policy formulation.

## Conclusion

Promoting energy transition is a common global trend in the context of resource constraints and increasingly severe environmental problems. Promoting clean heating in rural areas is crucial for China to achieve its dual-carbon and low-carbon transition in energy consumption goals. Based on 2023 research data, we analyzed the influence of policy incentives on CHB among rural residents in northern China. We examined the mediating effects of value perception and conducted a robustness test. Our findings led to the following conclusions. First, a direct effects estimation showed that policy incentives exert a direct positive impact on CHB among residents of northern China. Moreover, distinct variations exist in the influence of different policy types (i.e., advocacy, demonstration, and subsidy policies) on rural residents’ CHB. Notably, subsidy policy is the most influential factor for encouraging CHB. Second, we found that policy incentives indirectly affect residents’ CHB through the mediating role of value perception. Value perception acts as a critical mediating factor in the relationship between policy incentives and CHB, with varying mechanisms observed for different policy types: advocacy policies enhance residents’ economic and social value perceptions, thereby promoting the adoption of clean heating methods; demonstration policies facilitate the adoption of clean heating by influencing economic, social, functional, and emotional value perceptions; and subsidy policies effectively promote CHB by enhancing residents’ economic and emotional value perceptions.

Based on a comprehensive analysis, our findings have several policy implications. First, the government should prioritize recognizing the pivotal role of policy incentives in promoting clean heating practices among rural residents. Establishing and continuously enhancing a robust clean heating compensation mechanism while ensuring the focused implementation of government incentives are essential actions. The government should tailor the measures to each region’s specific circumstances, considering diverse factors such as economics, resource conditions, and regional climates. In doing so, the government can effectively develop targeted and refined policy schemes to improve the efficacy of clean heating systems. Additionally, the government must ensure the prompt and efficient disbursement of subsidies and innovatively structure clean heating subsidization to demonstrate an understanding of diverse residents’ economic capacities. Implementing subsidies can optimize policies’ overall effectiveness. This approach can facilitate a seamless transition toward cleaner, more sustainable heating in rural areas, ultimately contributing to the larger goals of environmental preservation and sustainable development.

Second, in addition to focusing on subsidy policies, the government should prioritize advocacy and demonstration policies. The government can effectively convey the importance and benefits of clean heating to rural residents through diverse communication channels, such as radio, television, brochures, and social media. A supportive service mechanism that provides clear, accurate information regarding clean heating must be established to address rural residents’ questions and concerns. This can be achieved by providing hotlines, online platforms, and on-site consultation services to ensure that residents receive timely support and assistance. Furthermore, technical staff are needed to provide on-site guidance, training courses, and other tools to help residents comprehend the working principles, operational procedures, and maintenance requirements of clean heating technology. By effectively leveraging advocacy and demonstration policies, the government can mitigate the various challenges and obstacles related to the adoption of clean heating methods.

Lastly, to foster the adoption of clean heating, enhancing residents’ value perception through various means is crucial, as is acknowledging the perceived trade-off between the benefits and costs of clean heating. The government should consider local conditions and choose suitable clean heating technologies to continually enhance indoor comfort. Additionally, residents should be educated on the health impact of indoor air quality as this can increase their perception of the functional value of clean heating. Furthermore, efforts should be made to improve the insulation performance of self-constructed houses and reduce the energy consumption of heating equipment to minimize economic costs. Simultaneously promoting energy technology safety and ensuring energy supply security are essential for mitigating residents’ perceptions of technical safety and energy supply risks.

## Supporting information

S1 Dataset Data sources. (XLSX)

S2 Data Descriptive statistics for the interquartile range of the variables. (DOCX)
