# Supplementary material for: The impact of policy incentives and value perception on rural residents’ clean heating behavior: Evidence from northern China
Source: PLoS One. 2025 Apr 17;20(4):e0321936. doi: 10.1371/journal.pone.0321936 (PMC12005538; doi:10.1371/journal.pone.0321936)
Supplement: S2 Data — (DOCX) [file pone.0321936.s002.docx]

**Table A1. Definitions of variables and descriptive statistics**

| Variables | Definition | Q25 | Q50 | Q75 |
| --- | --- | --- | --- | --- |
| Clean heating behavior | Whether rural residents adopted clean heating behavior (yes = 1, no = 0) | 0.000 | 1.000 | 1.000 |
| Advocacy policy | Whether the government promotes clean heating behavior (yes = 1, no = 0) | 0.000 | 1.000 | 1.000 |
| Demonstration policy | Whether the village has a clean heating demonstration project (yes = 1, no = 0) | 0.000 | 1.000 | 1.000 |
| Subsidy policy | Whether residents receive clean heating subsidies (yes = 1, no = 0) | 0.000 | 1.000 | 1.000 |
| Value perception | Whether residents perceive economic, social, functional, and emotional value (1= Strongly disagree, 2 = Disagree, 3 = Not sure, 4 = Agree, 5 = Strongly agree) | 0.619 | 0.750 | 0.830 |
| Gender | Resident’s gender (male = 1, female = 0) | 0.000 | 0.000 | 1.000 |
| Age | Resident’s age (1 = 18–25 years, 2 = 26–45 years, 3 = 46–65 years; 4 = 65 years and above) | 1.000 | 2.000 | 3.000 |
| Education level | Resident’s highest education level (1 = elementary school and below; 2 = junior high school; 3 = high school; 4 = college and above) | 3.000 | 4.000 | 4.000 |
| Social network | Number of cellphone contacts (1 = 0–29, 2 = 30–59, 3 = 60–99, 4 = 100–149, 5 =150–199, 6 = 200 and above) | 2.000 | 3.000 | 5.000 |
| Family size | Number of family members in household (1 = 1–2, 2 = 3–4, 3 = 5–6, 4 = 7 and above) | 2.000 | 2.000 | 2.000 |
| Heating area | Household winter heating area (1 = less than 30$m^{2}$; 2 = 30–60$m^{2}$, 3 = 60–90$m^{2}$, 4 = 90–110$m^{2}$, 5 = 110$m^{2}$and above) | 2.000 | 3.000 | 4.000 |
| Cadre | Whether someone in the family is a village cadre (yes = 1, no = 0) | 0.000 | 0.000 | 0.000 |
| Non-farm employment | Whether the family includes laborers working outside the home (yes = 1, no = 0) | 0.000 | 1.000 | 1.000 |
| Household income | Annual household income (1 = less than 10,000; 2 = 10,000–30,999; 3 = 40,000–79,999; 4 = 80,000–119,999; 5 = 120,000–159,999; 6 = 160,000 and above) | 2.000 | 3.000 | 4.000 |
| Neighborhood effect | Whether neighbors implement clean heating behavior (yes = 1, no = 0) | 0.000 | 1.000 | 1.000 |
